# Efficacy of Antiviral Treatment in Liver Biopsy-Proven Immune-Tolerant Chronic Hepatitis B Patients: A Retrospective Cohort Study

**DOI:** 10.3389/fmed.2021.655530

**Published:** 2021-04-08

**Authors:** Na Liu, Nan Yang, Wenqi Ma, Shujuan Yang, Chunhua Hu, Juan Li, Yingren Zhao, Guanghua Xu, Yingli He

**Affiliations:** ^1^Department of Infectious Diseases, The First Affiliated Hospital of Xi'an Jiaotong University, Xi’an, China; ^2^Shaanxi Clinical Research Center for Infectious Diseases, Xi’an, China; ^3^Department of Infectious Diseases, Yanan University Affiliated Hospital, Yan’an, China; ^4^Department of Ultrasound, The Second Affiliated Hospital of Xi'an Jiaotong University, Xi’an, China; ^5^Department of Infectious Diseases, The Eighth Hospital of Xi’an, Xi’an, China

**Keywords:** chronic hepatitis B, immune tolerance, antiviral treatment, virological response, liver stiffness

## Abstract

The optimal timing of initiating antiviral treatment for immune-tolerant (IT) patients remains unknown. We conducted this study in liver biopsy-proven IT patients to compare the long-term outcomes of untreated and treated patients suffering non-cirrhotic chronic hepatitis B (CHB). This retrospective cohort study recruited 171 consecutive treatment-naïve CHB patients who completed liver biopsy test. Patients were stratified into IT (*n* = 60), mildly-active (MA; *n* = 31), immune-active (IA; *n* = 80), according to alanine aminotransferase (ALT) and liver biopsy data. One hundred and nine patients receiving antiviral treatment constituted the treated set, and 62 patients under close follow-up comprised the untreated set. Primary outcomes were virological response, HBeAg seroconversion, HBsAg loss, ALT normalization, and liver stiffness measurement normalization (NCT03740789). The study population was predominantly male (62.6%) with a mean age of 31 years. The proportion of virological response in treated patients in the MA phase was 57.1%, and the proportion of HBeAg seroconversion was 28.6%, which showed no difference with the 43.8% virological response and 31.5% HBeAg seroconversion in IA patients. The proportions of virological response and seroconversion were 18.2 and 9.1%, respectively, in the IT phase, which were lower than the rates in the MA and IA phases. However, 95.5% of IT patients persisted normal ALT, and 100% of IT patients persisted normal liver stiffness measurement in the treated group. Therefore, antiviral treatment should be considered for CHB patients with high viral load regardless of phase to minimize further damage to hepatocytes.

## Introduction

Infection with hepatitis B virus (HBV) remains an important public health problem, not only in Asian countries, but also in Western countries (1). Chronic hepatitis B (CHB) is a life-threatening liver disease that causes cirrhosis and hepatocellular carcinoma (HCC), accounting for ~600,000 deaths per year worldwide (2, 3). The immune-tolerant (IT) phase is the first phase of CHB, characterized by high hepatitis B virus (HBV) DNA, positive HBeAg, normal alanine aminotransferase (ALT) and no or minimal liver damage (4). Antiviral treatment is not recommended under current guidelines because of the notion that the histological activity is dormant and the chance of serologic response is low in the IT phase (5–7). However, recent studies have sparked debate on the optimal timing of initiating antiviral treatment for IT patients. One retrospective study with a large sample number and one double-blind, randomized controlled trial revealed that antiviral treatment could benefit IT patients with mildly elevated or normal ALT (8, 9). Moreover, studies found that high HBV DNA levels were associated with high risks of HCC and cirrhosis, and that earlier antiviral treatment could prevent CHB-related mortality in IT patients (10).

These findings suggest that therapeutic interventions to minimize further damage to hepatocytes should be considered for IT-phase patients. However, most studies have used serum ALT to identify patients with necroinflammatory activity or fibrosis. Recent studies revealed that serum ALT level correlated poorly with the degree of liver disease in CHB. In a histologic series of 73 HBeAg-positive patients with persistently normal ALT levels, 40% of patients demonstrated significant liver fibrosis on liver biopsy (11). In another series using FibroScan among 243 HBeAg-positive patients with normal ALT, 35% had liver stiffness suggestive of advanced liver fibrosis (12). Conceivably, the treatment efficacy was exaggerated if patients with persistently normal ALT were considered as patients in the IT phase of for recruitment.

Liver biopsy is the gold standard for evaluating the histological severity of hepatic inflammation and fibrosis in CHB (6). Therefore, we investigated the antiviral treatment efficacy in liver biopsy-proven IT patients. We compared the long-term outcomes of treated and untreated patients in the IT phase and mildly active (MA) phases, which were excluded for treatment recommendations by the current practice guidelines. We also evaluated the kinetics of liver stiffness by FibroScan in these real-world practice settings.

## Materials and Methods

### Study Design and Patients

**Figure 1:**
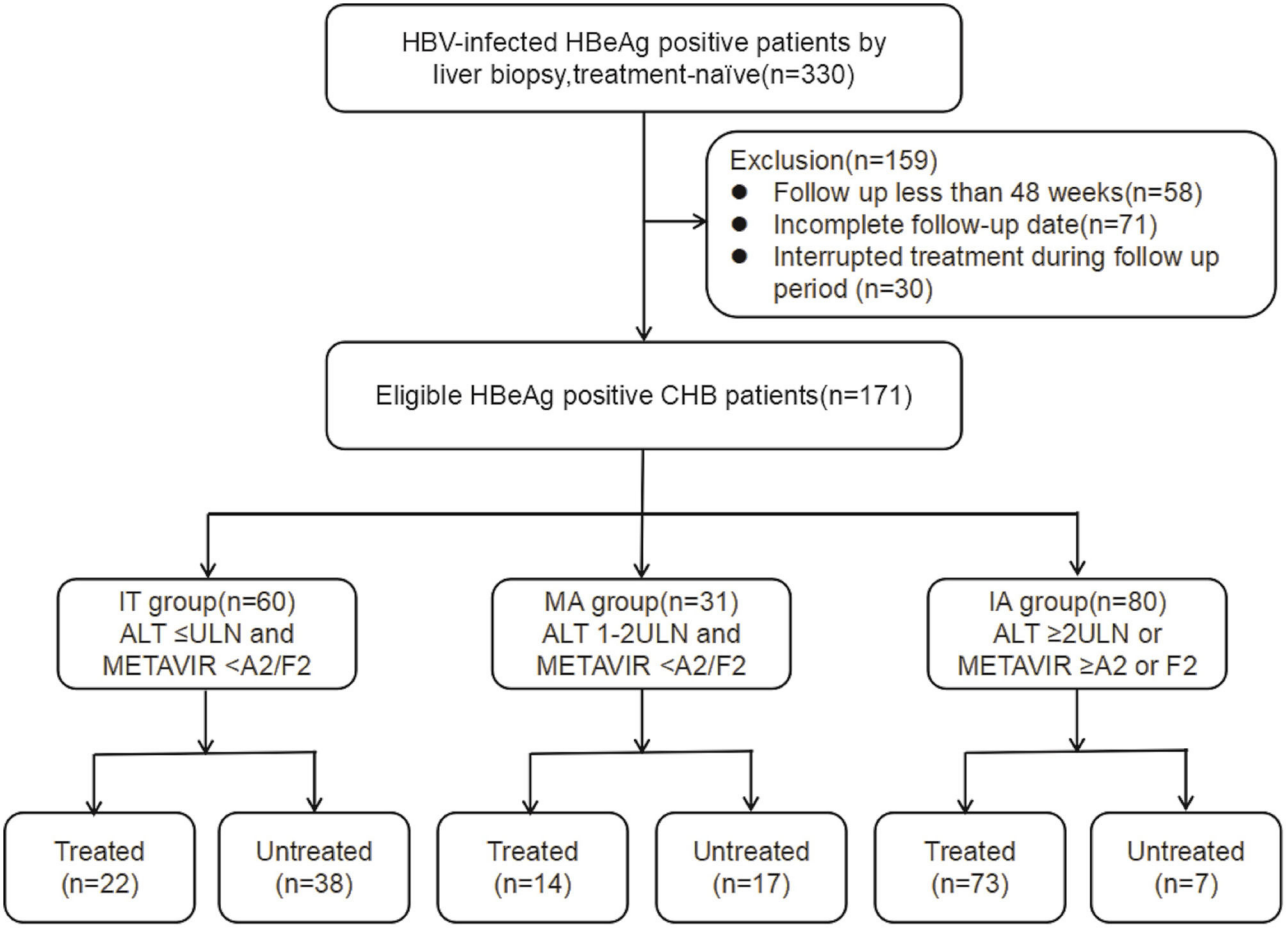
Flowchart showing enrollment of study patients. IT, immune tolerant; MA, mildly active; IA, immune active; CHB, chronic hepatitis; AST, aspartate aminotransferase; ULN, upper limit of normal.

This retrospective cohort study recruited 330 consecutive treatment-naïve CHB patients who completed liver biopsy test at the Yanan University Affiliated Hospital from 2013 to 2018. One hundred and fifty-nine patients were excluded, and 171 patients consequently comprised the study cohort (Figure 1). Patients were categorized into three CHB phases according to serum ALT level (5), and necroinflammatory and fibrosis stage indicated by liver biopsy (13). These three CHB phases were defined as follows: IT phase, ALT level ≤upper limit of normal (ULN), and METAVIR score <A2 and F2; MA phase, ULN <ALT level <2 × ULN, and METAVIR score <A2 and F2; immune active (IA) phase, ALT level ≥2 × ULN, or METAVIR score ≥A2 or F2. Patients were divided into two sets: patients receiving antiviral treatment constituted the treated set (*n* = 109) and patients under close follow-up constituted the untreated set (*n* = 62).

Treatment-naïve patients were orally treated with TDF (tenofovir disoproxil fumarate) 300 mg daily, ETV (entecavir) 0.5 mg daily, ADV (adefovir) 10 mg daily, LdT (telbivudine) 600 mg daily or LAM (lamivudine) 100 mg daily. Patients were given as percutaneous injections with pegylated interferon α-2a (Peg-IFN-α-2a) 180 μg once weekly after obtaining informed consent and excluding contraindications.

The primary efficacy endpoints were the proportions of patients with virological response, HBeAg seroconversion, HBsAg loss, ALT normalization, and liver stiffness measurement (LSM) normalization. Secondary efficacy endpoints included the proportions of patients with partial virological response (2 log_10_IU/mL or greater decrease in HBV DNA), 0.5 log_10_IU/mL or greater decrease in HBsAg, 1 log_10_IU/mL or greater decrease in HBeAg, and LSM improvement (one kPa or greater decrease in LSM).

The study was carried out in accordance with the guidelines of the 1983 Declaration of Helsinki and was approved by the Institutional Ethics Committee of the First Affiliated Hospital of Xi'an Jiaotong University and the Yan'an University Affiliated Hospital. Written informed consent was obtained from all subjects. This study was registered at www.clinicaltrials.gov as NCT03740789.

### Liver Biopsy

Liver samples were obtained using 16-gauge Menghini needles with ultrasound localization. Biopsies were required to be more than 15 mm in length and to include at least 12 portal areas. The grading of inflammation and fibrosis staging of liver biopsy specimens was analyzed according to the METAVIR scoring system by experienced pathologists blinded to patients' clinical information.

### Liver Stiffness Measurement

LSM was performed using the FibroScan^®^ 502 Touch system equipped with the standard probe (Echosens, Paris, France) according to the instructions by experienced physicians. Reliable transient elastography measurements were defined as median values of 10 valid LSM, with interquartile range <30% and success rate ≥60% (13). Liver fibrosis stages F0–F4 according to LSM were set as follows: no/little fibrosis (F0/F1), <7.3 kPa; significant fibrosis (F2), ≥7.3 and <9.7 kPa; advanced fibrosis (F3), ≥9.7 and <12.4 kPa; cirrhosis (F4), ≥12.4 kPa.

### Statistical Analysis

The data were analyzed using SPSS version 20.0 (SPSS, Chicago, IL). The data are presented as the mean or median for quantitative data and as proportions for categorical data. Differences between two subgroups were analyzed using the χ^2^-test for categorical parameters or Mann-Whitney *U*-test for continuous parameters. For multiple subgroups, differences were evaluated using ANOVAs or Kruskal-Wallis tests. Univariable and multivariable analyses were used to determine predictors of virological response. All statistical tests were two-tailed, and *P* < 0.05 was considered statistically significant.

## Results

### Characteristics of the Study Population

The primary study population comprised 60 IT-phase, 31 MA-phase, and 80 IA-phase patients with median follow-up of 96 weeks (range, 48–144 weeks). The study population was predominantly male (62.6%) with a mean age of 31 years. All patients were of Asian ethnicity. Almost all were infected with genotype C (97.1%), and the majority had a family history of hepatitis B (66.7%). Nucleos(t)ide analogs (NAs) initially administered to 91 (83.5%) treated patients comprised ETV (*n* = 55, 50.5%), TDF (*n* = 6, 5.5%), other NAs (*n* = 30, 27.5%). Peg-IFN-α-2a initially administered to 18 (16.5%) treated patients. All these characteristics were comparable among the three CHB phases (*P* > 0.05, Table 1). Defined as advanced necroinflammatory and fibrosis stage, IA patients had higher levels of ALT and LSM than IT and MA patients (mean ALT 65 U/L vs. 23 U/L and 39 U/L, respectively; mean LSM 6.8 kPa vs. 4.8 kPa and 5.0 kPa, respectively; *P* < 0.05; Table 1).

**Table 1:** Baseline characteristics in liver biopsy-proven IT-, MA-, and IA-phase patients.

	**Study population**	**IT phase**	**MA phase**	**IA phase**	***P***
	**(*n* = 171)**	**(*n* = 60)**	**(*n* = 31)**	**(*n* = 80)**	
Age (years)	31 (26–40)	30 (26–40)	31 (26–41)	32 (25–38)	0.911
Male	107 (62.6%)	39 (65.0%)	17 (54.8%)	51 (63.8%)	0.610
Family history	114 (66.7%)	43 (71.7%)	20 (64.5%)	51 (63.8%)	0.593
Genotype C	166 (97.1%)	58 (96.7%)	30 (96.8%)	78 (97.5%)	0.953
HBV DNA (log_10_ IU/ml)	7.54 (6.80–8.12)	7.68 (7.34–8.27)	7.67 (6.89–8.36)	7.14 (6.03–7.97)	0.001
≥7 log_10_ IU/ml	120 (70.2%)	55 (91.7%)	22 (71.0%)	43 (53.75%)	
HBsAg (log_10_ IU/ml)	4.33 (3.72–4.71)	4.69 (4.45–4.91)	4.38 (3.98–4.73)	3.85 (3.39–4.31)	<0.001
HBeAg (log_10_ S/CO)	3.06 (2.01–3.16)	3.13 (3.09–3.19)	3.12 (2.85–3.18)	2.67 (1.63–3.02)	<0.001
HBeAb (log_10_ S/CO)	1.66 (0.76–1.76)	1.73 (1.66–1.81)	1.73 (1.57–1.77)	1.32 (0.44–1.66)	<0.001
HBcAb (S/CO)	9.72 (8.14–10.96)	8.39 (7.07–10.20)	10.30 (8.71–10.90)	10.29 (9.50–11.92)	<0.001
ALT (U/L)	36 (24–63)	23 (18–27)	39 (34–48)	65 (41–96)	<0.001
≤1 × ULN	67 (39.2%)	60 (100%)	0 (0%)	7 (8.75%)	
ULN-2 × ULN	58 (33.9%)	0 (0%)	31 (100%)	27 (33.75%)	
≥2 × ULN	46 (26.9%)	0 (0%)	0 (0%)	46 (57.5%)	
AST (U/L)	29 (21–46)	20 (18–24)	30 (24–35)	46 (31–62)	<0.001
TB (mg/dL)	0.76 ± 0.36	0.72 ± 0.28	0.73 ± 0.39	0.81 ± 0.39	0.338
ALB (g/L)	41.98 ± 4.70	42.20± 4.20	41.56 ± 3.89	41.97 ± 5.34	0.828
LSM (kPa)	5.6 (4.5–6.9)	4.8 (4.0–5.6)	5.0 (4.2–6.1)	6.8 (5.4–9.0)	<0.001
<7.3 kPa	133 (77.8%)	59 (98.3%)	29 (93.5%)	45 (56.25%)	
Anti-viral treatment	109	22	14	73	
NAs	91 (83.5%)	18 (81.8%)	11 (64.3%)	62 (84.9%)	0.533
ETV	55 (50.5%)	8 (36.4%)	8 (28.6%)	39 (53.4%)	
TDF	6 (5.5%)	0 (0%)	1 (7.1%)	5 (6.8%)	
Other NAs	30 (27.5%)	10 (45.4%)	2 (28.6%)	18 (24.7%)	
Peg-IFN-α-2a	18 (16.5%)	4 (18.2%)	3 (35.7%)	11 (15.1%)	

*IT, immune tolerant; MA, mildly active; IA, immune active; HBV, hepatitis B virus; HBsAg, HBV surface antigen; HBeAg, HBV e antigen; HBeAb, HBV e antibody; HBcAb, HBV core antibody; ALT, alanine aminotransferase; ULN, upper limit of normal; AST, aspartate aminotransferase; TB, total bilirubin; ALB, albumin; LSM, liver stiffness measurement; NAs, nucleos(t)ide analogs; ETV, entecavir; TDF, tenofovir disoproxil fumarate; Peg-IFN-α-2a, pegylated interferon α-2a*.

### Primary Efficacy Endpoints

#### Virological Response

To illuminate the benefit of antiviral treatment, we performed analysis between treated and untreated groups. Forty-four of 109 (40.4%) treated patients achieved virological response compared with none in the untreated group (Table 2). The proportion achieving virological response in treated IT, MA, and IA patients were 18.2, 57.1, and 43.8%, respectively, which were all significantly higher than the proportion in corresponding untreated groups (*P* < 0.05, Table 2). Both univariate and multivariate analyses showed that family history and baseline anti-HBc level were significantly associated with virological response in IT and MA treated groups (*P* < 0.05, Table 3).

**Table 2:** Efficacy endpoints in liver biopsy-proven IT, MA, and IA-phase patients.

	**IT phase**			**MA phase**			**IA phase**	
	**Treated (*n* = 22)**	**Untreated (*n* = 38)**	***P***	**Treated (*n* = 14)**	**Untreated (*n* = 17)**	***P***	**Treated (*n* = 73)**	**Untreated (*n* = 7)**	***P***
**Primary outcomes**									
Virological response	4 (18.2%)	0 (0%)	0.015	8 (57.1%)	0 (0%)	<0.001	32 (43.8%)	0 (0%)	0.038
HBeAg seroconversion	2 (9.1%)	0 (0%)	0.131	4 (28.6%)	0 (0%)	0.032	23 (31.5%)	0 (0%)	0.184
Persistence of normal ALT	21 (95.5%)	23 (60.5%)	0.003	11 (78.6%)	3 (17.6%)	0.001	48 (65.8%)	0 (0%)	0.001
Persistence of normal LSM	22 (100%)	33 (86.8%)	0.148	14 (100%)	12 (70.6%)	0.048	62 (84.9%)	2 (28.6%)	0.003
**Secondary outcomes**									
Partial virological response	10 (45.5%)	2 (5.3%)	<0.001	5 (35.7%)	2 (11.8%)	0.248	32 (43.8%)	1 (14.3%)	0.265
HBsAg decline ≥0.5 log_10_IU/mL	9 (40.9%)	1 (2.6%)	<0.001	6 (42.9%)	0 (0%)	0.004	18 (24.7%)	4 (57.1%)	0.163
HBeAg decline ≥1 log_10_S/CO	6 (27.3%)	1 (2.6%)	0.008	6 (42.9%)	0 (0%)	0.004	39 (53.4%)	2 (28.6%)	0.258
LSM improvement	6 (27.3%)	1 (2.6%)	0.008	7 (50.0%)	1 (5.9%)	0.011	47 (64.4%)	0 (0%)	0.001

*Virological response was defined as HBV DNA <lower limit of quantification. HBeAg seroconversion was defined as the HBeAg loss and appearance of HBeAb. Persistence of normal ALT was defined as ALT level ≤35 U/L for males and ≤25 U/L for females. Persistence of normal LSM was defined as LSM <7.3 kPa. Partial virological response was defined as 2 log_10_IU/mL or greater decrease of HBV DNA. LSM improvement was defined as one kPa or greater decrease of LSM*.

*IT, immune tolerant; MA, mildly active; IA, immune active; HBeAg, HBV e antigen; ALT, alanine aminotransferase; LSM, liver stiffness measurement; HBsAg, HBV surface antigen*.

**Table 3:** Factors predictive of virological response in liver biopsy-proven IT and MA-phase patients.

	**Univariate analysis**		**Multivariate analysis**	
	**HR (95% CI)**	***P***	**HR (95% CI)**	***P***
Age (years)	2.000 (0.486–8.229)	0.337		
Male	0.061 (0.006–0.612)	0.017		
Family history	0.143 (0.030–0.688)	0.015	0.060 (0.006–0.626)	0.019
Baseline HBV DNA (log_10_ IU/ml)	0.647 (0.382–1.096)	0.105		
Baseline HBsAg (log_10_ IU/ml)	0.170 (0.037–0.773)	0.022		
Baseline HBeAg (log_10_ S/CO)	0.272 (0.090–0.824)	0.021		
Baseline HBcAb (S/CO)	1.943 (1.156–3.265)	0.012	2.426 (1.269–4.639)	0.007
Baseline ALT (U/L)	1.010 (0.951–1.073)	0.748		
Baseline AST (U/L)	1.006 (0.931–1.087)	0.883		
Baseline TB (μmol/L)	0.980 (0.872–1.100)	0.729		
Baseline ALB (g/L)	0.778 (0.610–0.993)	0.044		
Baseline LSM (kPa)	1.053 (0.664–1.671)	0.825		

*IT, immune tolerant; MA, mildly active; HR, hazard ration; CI, confidence interval; HBV, hepatitis B virus; HBsAg, HBV surface antigen; HBeAg, HBV e antigen; HBcAb, HBV core antibody; ALT, alanine aminotransferase; AST, aspartate aminotransferase; TB, total bilirubin; ALB, albumin; LSM, liver stiffness measurement*.

#### HBeAg Seroconversion and HBsAg Loss

Twenty-nine of 109 (26.6%) treated patients achieved HBeAg seroconversion compared with none in the untreated set (Table 2). The proportion of HBeAg seroconversion in treated IT, MA, and IA patients were 9.1, 28.6, and 31.5%, respectively (Table 2). Only baseline anti-HBc was significantly associated with HBeAg seroconversion in IT and MA treated groups (*P* < 0.05, Table 4), and just one patient achieved HBsAg loss, belonging to the treated IA group.

**Table 4:** Factors predictive of HBeAg seroconversion in liver biopsy-proven IT and MA-phase patients.

	**Univariate analysis**		**Multivariate analysis**	
	**HR (95% CI)**	***P***	**HR (95% CI)**	***P***
Age (years)	1.067 (0.240–4.740)	0.932		
Male	0.727 (0.111–4.768)	0.740		
Family history	1.037 (0.212–5.077)	0.964		
Baseline HBV DNA (log_10_ IU/ml)	0.824 (0.511–1.327)	0.426		
Baseline HBsAg (log_10_ IU/ml)	0.443 (0.124–1.579)	0.209		
Baseline HBeAg (log_10_ S/CO)	0.769 (0.321–1.839)	0.555		
Baseline HBcAb (S/CO)	1.540 (1.004–2.363)	0.048	1.540 (1.004–2.363)	0.048
Baseline ALT (U/L)	1.040 (0.976-1.109)	0.227		
Baseline AST (U/L)	0.956 (0.867–1.054)	0.367		
Baseline TB (μmol/L)	1.043 (0.927–1.175)	0.481		
Baseline ALB (g/L)	0.868 (0.689–1.094)	0.231		
Baseline LSM (kPa)	0.774 (0.451–1.330)	0.354		

*IT, immune tolerant; MA, mildly active; HR, hazard ration; CI, confidence interval; HBV, hepatitis B virus; HBsAg, HBV surface antigen; HBeAg, HBV e antigen; HBcAb, HBV core antibody; ALT, alanine aminotransferase; AST, aspartate aminotransferase; TB, total bilirubin; ALB, albumin; LSM, liver stiffness measurement*.

#### ALT Normalization

Of the IT patients, 95.5% persisted normal ALT in the treated group, while 39.5% of untreated IT patients showed ALT deterioration. 78.6% had normal ALT levels after antiviral treatment, while three (17.6%) patients underwent spontaneous ALT normalization in the untreated group. The proportion of ALT normalization in treated group of IA patients was 65.8%, compared with none (0%) in the untreated group. Compared with the untreated set, treated groups had higher proportions of ALT normalization regardless CHB phase (*P* < 0.05, Table 2).

#### LSM Normalization

All IT and MA patients persisted normal LSM in the treated group, while 13.2 and 29.4% of untreated IT and MA patients, respectively, demonstrated LSM deterioration (Table 2). In the IA phase, 84.9% of patients had normal LSM values after antiviral treatment, while two (28.6%) patients underwent spontaneous LSM normalization in the untreated group (*P* < 0.05, Table 2).

### Secondary Efficacy Endpoints

#### Partial Virological Response

The proportions of partial virological response in treated IT, MA and IA patients were 45.5, 35.7, and 43.8%, respectively (Table 2). However, only two IT patients, two MA patients and one IA patient achieved partial virological response spontaneously (Table 2).

#### HBsAg Decrease

The proportion of HBsAg decline ≥0.5 log_10_IU/mL in treated groups of IT, MA, and IA patients were 40.9, 42.9, and 24.7%, respectively (Table 2). However, only one IT patient, no MA patients and four IA patients achieved HBsAg decline ≥0.5 log_10_IU/mL spontaneously (Table 2).

#### HBeAg Decrease

The proportions of HBeAg decline ≥1 log_10_S/CO in treated IT, MA, and IA patients were 27.3, 42.9, and 53.4%, respectively (Table 2). However, only one IT patient, no MA patient and two IA patients achieved HBeAg decline ≥1 log_10_S/CO spontaneously (Table 2).

#### LSM Improvement

The proportions of LSM decline ≥1 kPa in treated IT, MA, and IA patients were 27.3, 50.0, and 64.4%, respectively (Table 2). However, only one IT patient, one MA patient and no IA patients achieved LSM decline ≥1 kPa spontaneously (Table 2).

### Dynamics of Clinical Indices From Baseline to Endpoint

**Figure 2:**
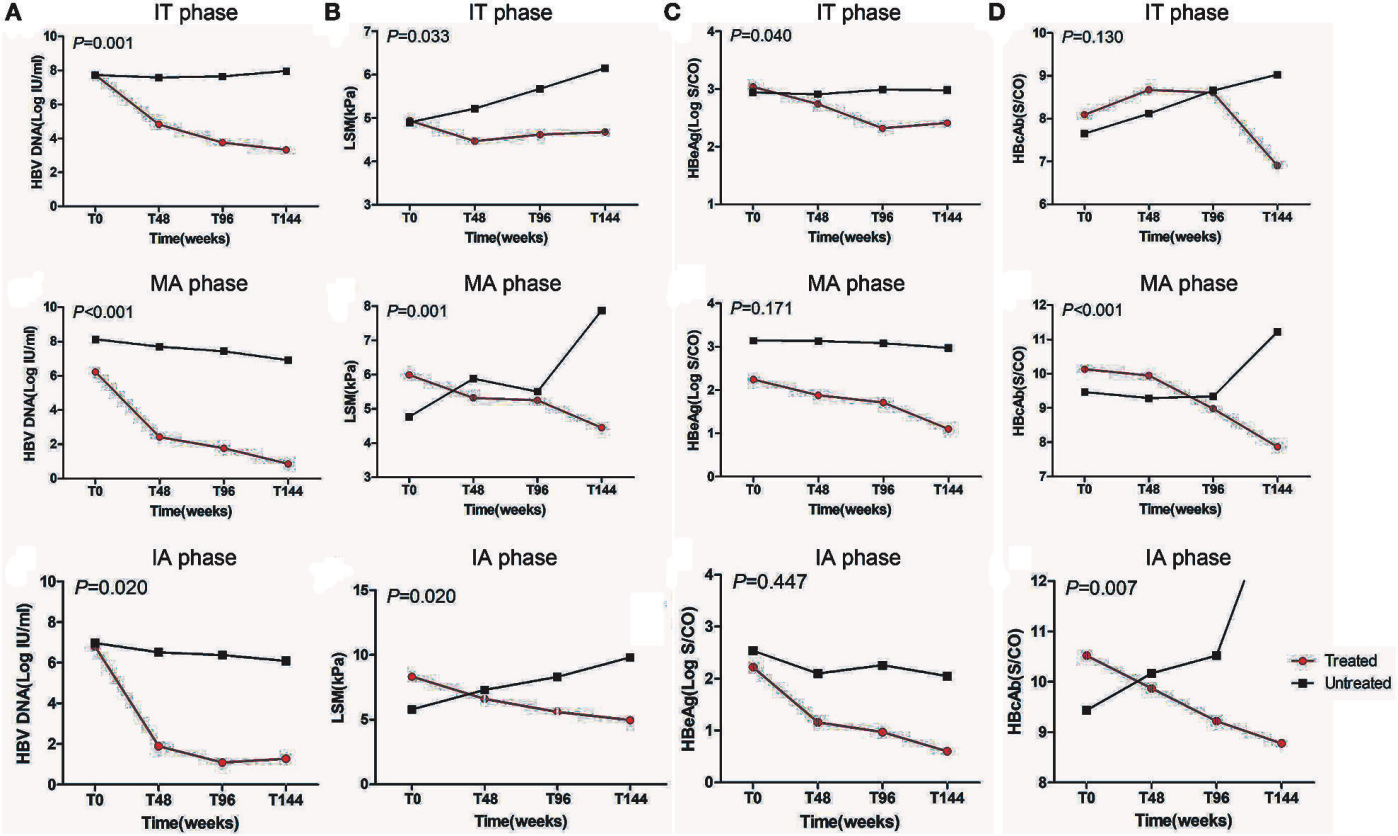
Dynamics of HBV DNA, HBeAg levels, anti-HBc levels, and liver stiffness values from baseline to endpoint. IT, immune tolerant; MA, mildly active; IA, immune active; LSM, liver stiffness measurement.

HBV DNA significantly decreased after antiviral treatment regardless of CHB phases compared with untreated patients (*P* < 0.05, Figure 2A). LSM values remained stable after antiviral treatment in the IT phase and significantly improved in MA and IA phases compared with untreated patients in corresponding phases (*P* < 0.05, Figure 2B). Furthermore, the treated IT group showed a significant decrease in HBeAg levels compared with the corresponding untreated group (*P* < 0.05, Figure 2C). In the MA and IA phases, there was a significant decrease in anti-HBc level in the treated set compared with untreated patients (*P* < 0.05, Figure 2D).

**Figure 3:**
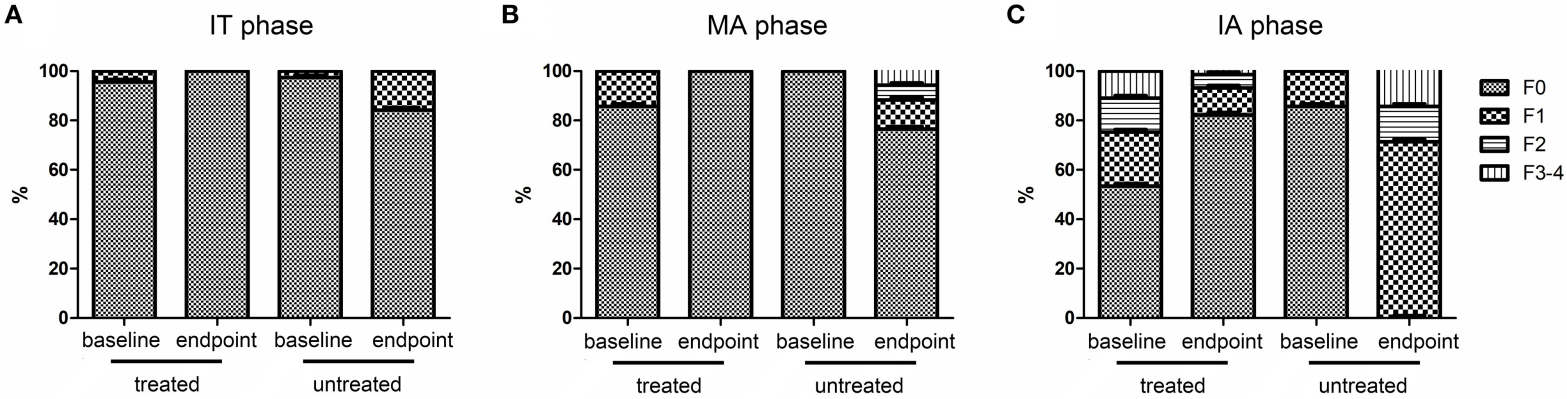
Dynamics of fibrosis stage indicated by FibroScan from baseline to endpoint. IT, immune tolerant; MA, mildly active; IA, immune active.

HBV DNA levels significantly declined from baseline to endpoint in treated groups (*P* < 0.05, Table 5) but remained high in untreated groups regardless of CHB phase (*P* > 0.05, Table 5). Similarly, HBsAg and HBeAg levels significantly declined from baseline to endpoint in the treated set (*P* < 0.05, Table 5) but remained high in untreated patients (*P* > 0.05, Table 5). ALT level and LSM value remained normal, and there were no differences from baseline to endpoint in treated IT patients (*P* > 0.05, Table 5 and Figure 3A). However, ALT levels and LSM values significantly decreased from baseline to endpoint in treated MA and IA groups (*P* > 0.05, Table 5 and Figures 3B,C). Conversely, significantly elevated ALT and LSM were recorded from baseline to endpoint in untreated groups of both IT and MA patients (*P* < 0.05, Table 5 and Figures 3A,B). Consistent with the primary and secondary outcomes, the dynamics of HBV DNA, HBsAg, HBeAg, ALT, and LSM values from baseline to endpoint revealed that antiviral treatment was beneficial to patients with virological and serological improvement, and could prevent biochemical and histological deterioration in IT- and MA-phase patients.

**Table 5:** Comparison of outcomes from baseline to endpoint.

	**HBV DNA (log**_****10****_ **IU/ml)**			**HBsAg (log**_****10****_ **IU/ml)**			**HBeAg (log**_**10**_ **S/CO)**			**HBeAb (log**_**10**_ **S/CO)**			**HBcAb (S/CO)**			**ALT (U/L)**			**AST (U/L)**			**LSM (kPa)**		
	**Baseline**	**Endpoint**	***P***	**Baseline**	**Endpoint**	***P***	**Baseline**	**Endpoint**	***P***	**Baseline**	**Endpoint**	***P***	**Baseline**	**Endpoint**	***P***	**Baseline**	**Endpoint**	***P***	**Baseline**	**Endpoint**	***P***	**Baseline**	**Endpoint**	***P***
**IT phase**																								
Treated group	7.65 (7.38–8.00)	2.98 (1.99–7.11)	<0.001	4.57 (4.38–4.89)	4.17 (3.51–4.65)	0.008	3.11 (3.04–3.20)	2.99 (1.56–3.13)	0.002	1.74 (1.64–1.85)	1.64 (0.48–1.71)	0.009	8.09 ± 3.30	7.98 ± 2.78	0.906	24.50 ± 5.65	25.00 ± 4.94	0.756	23.91 ± 9.72	23.27 ± 4.00	0.778	4.94 ± 1.31	4.67 ± 0.96	0.436
Untreated group	7.74 ± 1.09	7.85 ± 1.42	0.698	4.59 ± 0.53	4.60 ± 0.53	0.961	2.94 ± 0.61	3.01 ± 0.54	0.616	1.57 ± 0.60	1.53 ± 0.69	0.807	7.65 ± 2.84	8.23 ± 2.18	0.322	20.87 ± 6.06	30.95 ± 25.56	0.021	21.68 ± 7.44	31.08 ± 20.10	0.009	4.90 ± 1.05	5.72 ± 1.27	0.003
**MA phase**																								
Treated group	6.23 ± 1.90	1.15 ± 1.60	<0.001	4.04 ± 0.70	3.49 ± 0.62	0.037	2.24 ± 1.05	1.10 ± 1.37	0.020	1.04 ± 0.93	0.13 ± 1.25	0.039	10.13 ± 1.45	8.09 ± 1.17	<0.001	41.79 ± 10.37	27.21 ± 16.23	0.009	28.71 ± 6.90	23.43 ± 9.38	0.101	5.99 ± 1.64	4.60 ± 0.97	0.011
Untreated group	8.14 ± 0.77	7.64 ± 1.96	0.340	4.39 ± 0.46	4.47 ± 0.49	0.655	3.14 ± 0.66	3.09 ± 0.15	0.179	1.73 ± 0.06	1.67 ± 0.13	0.062	9.47 ± 1.51	9.83 ± 2.17	0.580	40.88 ± 9.69	57.71 ± 29.06	0.035	31.00 ± 7.77	40.00 ± 13.30	0.022	4.77 ± 0.93	6.48 ± 3.14	0.044
**IA phase**																								
Treated group	6.80 ± 1.53	1.52 ± 1.91	<0.001	3.79 ± 0.61	3.52 ± 0.79	0.022	2.66 (1.54–3.00)	0.88 (−0.23–1.97)	<0.001	1.31 (0.44–1.65)	0.21 (−0.16–0.78)	<0.001	10.52 ± 1.57	9.07 ± 2.05	<0.001	64 (40–94)	25 (17–39)	<0.001	46 (31–60)	26 (22–34)	<0.001	6.9 (5.5–9.7)	5.1 (4.5–6.3)	<0.001
Untreated group	6.69 ± 2.24	6.32 ± 2.13	0.582	4.17 ± 0.77	3.66 ± 0.58	0.186	2.54 ± 1.15	1.12 ± 0.42	0.433	1.12 ± 0.80	0.91 ± 0.81	0.621	9.44 ± 2.12	12.24 ± 2.78	0.056	76.14 ± 35.99	60.71 ± 25.79	0.375	52.29 ± 20.32	45.29 ± 9.25	0.423	5.79 ± 1.40	8.64 ± 2.25	0.015

*IT, immune tolerant; MA, mildly active; HBV, hepatitis B virus; HBsAg, HBV surface antigen; HBeAg, HBV e antigen; HBeAb, HBV e antibody; HBcAb, HBV core antibody; ALT, alanine aminotransferase; AST, aspartate aminotransferase; LSM, liver stiffness measurement*.

## Discussion

HBV infection remains an important global public health problem (1) and IT-phase patients constitute a large proportion of CHB patients because vertical transmission is the principal route of HBV infection (2). Emphasizing that treatment should be initiated when serum ALT exceeds ULN or there is evidence of histological damage, current guidelines exclude IT patients for treatment recommendations. However, even among patients with persistently normal ALT levels, 28–60% may have significant hepatic necroinflammation and/or fibrosis (10). Considering the risk of overestimated efficacy in ALT-confirmed IT patients, we conducted this retrospective cohort study in liver biopsy-proven IT patients.

We compared efficacy outcomes in treated and untreated patients in IT-, MA-, and IA phases. To our great surprise, similar treatment responses were observed between patients in MA and IA phases. The proportion of patients achieving virological response in the treated MA phase group was 57.1% while the proportion of HBeAg seroconversion was 28.6%, showing no difference from the 43.8% virological response and 31.5% HBeAg seroconversion observed in treated IA phase patients. In a previous study of pediatric IT phase (age 1–16 years) by Zhu et al. (14), 73.9% of patients became HBV DNA negative, 32.6% achieved HBeAg seroconversion and 21.7% achieved HBsAg loss at week 96. Another study of adult IT phase (age 18–69 years) by Chan et al. (9) showed that 54.7% of patients demonstrated levels of HBV DNA <69 IU/mL, 5.8% underwent HBeAg seroconversion and no patient had loss of HBsAg at week 192. Consistent with the previous studies, the proportion of virological response in our study was 18.2%, and the proportion of HBeAg seroconversion was 9.1% in the IT phase, which was relatively low compared with the MA and IA phases. However, antiviral treatment in IT and MA phases could maintain ALT and LSM normalization, preventing biochemical flare and histological deterioration, which may stop liver disease progression and HBV-related mortality.

Furthermore, we explored the predictive factors for virological response and HBeAg seroconversion in IT and MA patients. Univariate and multivariate analyses found that high baseline anti-HBc level was significantly correlated with both virological response and HBeAg seroconversion. A recent study showed that anti-HBc levels were associated with ALT levels and could distinguish active hepatitis B patients from inactive carriers (15), meaning that anti-HBc might also be a biomarker of inflammation or fibrosis for HBeAg positive patients (16, 17). Notably, anti-HBc levels showed a downward trend in treated patients compared with untreated patients in our study, all of which inferred that anti-HBc level could predict therapeutic response (18–21). The mechanism for the predictive value of anti-HBc was mediating the immune function of B-cell in chronic HBV infections (22).

During CHB infection, chronic inflammation can lead to fibrosis and cirrhosis, which is the background of HCC (23). LSM had been reported as a reliable non-invasive method for assessing the degree of liver fibrosis in CHB patients with ALT <2 × ULN (24). The decline in LSM in the treated subgroups in our study, to a certain extent, reflected the remission of histological lesions (25, 26). More importantly, histological response, in particular decreasing hepatic fibrosis meant a lower risk of development of cirrhosis and HCC in CHB patients (27). Overall, monitoring LSM changes during antiviral therapy is of strategic importance not only for predicting disease progress but also for evaluating histological response (28).

There were some limitations to our study. Firstly, this study was a retrospective investigation from a single center, and therefore the sample size was not large enough to fully reflect all patients. However, the data presented are persuasive because all patients in the cohort were from Asia, which ensured consistency of the sample. Secondly, owing to the short median follow-up, we could not draw any conclusion about long-term clinical outcomes such as the incidences of HCC or transplantation. However, a recent study has reported that antiviral therapy significantly reduced the risk of HCC in treated IT patients compared with untreated IT patients (29). Finally, although all patients underwent the first biopsy to precisely evaluate degree of liver fibrosis, the majority were reluctant to accept follow-up biopsy to evaluate dynamic changes. To minimize this shortcoming, LSM was measured by liver transient elastography at intervals of 3 or 6 months.

In conclusion, HBeAg-positive CHB patients with mildly elevated ALT and no or minimal histological damage showed similar responses to patients with ALT ≥2 × ULN or moderate-to-severe histological damage. CHB patients with normal ALT and no liver disease showed lower treatment response, but maintained biochemical and histological normalization. Antiviral treatment should be considered for CHB patients with high viral load regardless of phase to minimize further damage to hepatocytes and prevent HBV-related mortality.

## Data Availability Statement

The raw data supporting the conclusions of this article will be made available by the authors, without undue reservation.

## Ethics Statement

The studies involving human participants were reviewed and approved by the Institutional Ethics Committee of the First Affiliated Hospital of Xi'an Jiaotong University and Yan'an University Affiliated Hospital. The patients/participants provided their written informed consent to participate in this study. Written informed consent was obtained from the individual(s) for the publication of any potentially identifiable images or data included in this article.

## Author Contributions

YH and GX designed the study and revised the manuscript. NL and NY collected data, analyzed data, and drafted the manuscript. WM, SY, CH, and JL collected and analyzed data. YZ, YH, and GX reviewed the results and made critical comments on the manuscript. All authors contributed to the article and approved the submitted version.

## Conflict of Interest

The authors declare that the research was conducted in the absence of any commercial or financial relationships that could be construed as a potential conflict of interest.
